# PRR14 acts a novel oncogene activating the PI3K signal pathway in human cutaneous squamous cell carcinoma

**DOI:** 10.7150/jca.83695

**Published:** 2023-05-21

**Authors:** Lili Zhang, Jie Yang, Zhoujing Ji, Jie Zhang, Shengju Yang

**Affiliations:** 1 Department of Dermatology, Affiliated Hospital of Nantong University, Nantong, Jiangsu, China.; 2 Department of Dermatology, Tongzhou District Home Textile City Hospital, Nantong, Jiangsu, China.

**Keywords:** cutaneous squamous cell carcinoma, proline rich protein 14, PI3K signal pathway.

## Abstract

Proline rich protein 14 (PRR14) is considered as a new component of the nuclear fiber layer, it may be a key molecule in mediating nuclear morphological changes and functional changes in tumorigenesis. But, it's still unclear in human cutaneous squamous cell carcinoma (cSCC). In the study, the expression profiles of PRR14 in patients with cSCC were investigated by immunohistochemistry (IHC), also the PRR14 expression in cSCC tissues were detected using the methods of real-time quantitative PCR (RT-qPCR) and Western blot; cell counting kit-8 (CCK-8) assay, wound healing assay, matrigel-based transwell assay and Annexin V-FITC and PI double-staining with flow cytometry assay were used to investigate the biological functions of PRR14 in A431 and HSC-1 cSCC cells. Overexpression of PRR14 in cSCC patients was reported firstly in this study and its high expression was related to differentiation, thickness and tumor node metastasis (TNM) stage of cSCC. PRR14 inhibition with RNA interfering (RNAi) method resulted in the suppression of cell proliferation, migration and invasion but promotion the apoptosis of cSCC cells, and upregulation of the protein phosphorylation levels of mammalian target of rapamycin (mTOR), phosphoinositide 3-kinase (PI3K) and Akt. The study shows PRR14 maybe an activator of cSCC carcinogenesis through PI3K/Akt/mTOR signal pathway, and it also maybe a prognostic factor and new therapeutical target for cSCC treatment.

## Introduction

Human cutaneous squamous cell carcinoma (cSCC) main originates from epidermal keratinocytes, it is an invasive and potentially malignant cutaneous carcinoma, about 15% of malignant epithelial tumors 1. The pathogenesis of cSCC is complex, which might be correlation with ultraviolet radiation, chemicals exposure, precancerous dermatosis, skin trauma or scar and the application of immunosuppressant, but its specific pathogenesis is not clear 2. Therefore, the study of mechanism, prevention and therapy of recurrence and metastasis of cSCC is the main problem to be solved urgently in the dermatological field.

Nuclear morphological changes are the diagnosis standard for numerous cancers, and the common features of tumor cells are nucleus enlargement and obvious nucleoli 3. But, the involved specific nuclear components, underlying mechanisms and effects on tumor progression are still unclear. Nuclear fiber layer is a scaffold-like protein filament network around the nucleus, which is necessary for the regulation of numerous nuclear activities, i.e. DNA replication, RNA transcription, nuclear morphology, cell cycle regulation, nuclear and chromatin organization, cell differentiation and development 4. In the initial stage of tumorigenesis, the composition of nuclear lamina such as Lamin A/C has changed significantly, and acts biomarkers of certain cancers, including colorectal cancer 5, breast cancer 6 and lung cancer 7, as well as Lamin B1 in liver cancer 8. During the development of tumor, the nuclear fiber layer plays a critical role in the morphological and functional changes of connective nuclei 9, 10.

Proline rich protein 14 (PRR14) is a member of the proline rich proteins (PRRs) family, which involved a proline rich region with C-terminal and N-terminal nuclear localization signals (NLS) on both sides 11. Proline rich regions usually participate in a variety of signal pathways by binding to various domains (especially SH3 domain) 12, 13. PRR14 coded protein is considered to be a new component of the nuclear fiber layer, it has the function of connecting heterochromatin to the nuclear fiber layer, and endowing heterochromatin with specificity and localization during the cell cycle 14. During the process of cell cycle, PRR14 presents a dynamic distribution: it's released from the nuclear fiber layer and chromatin, and then remains soluble; at the beginning of the later stage of mitosis, PRR14 rapidly incorporates into chromatin; at the end of mitosis, it relocates to the recombinant nuclear fiber layer 14. PRR14 may be a key molecule in mediating nuclear morphological changes and functional changes in tumorigenesis. It has been reported that PRR14 was abnormally overexpressed in the tumor tissues of patients with non-small cell lung cancer (NSCLC), and related to poor prognosis, and PRR14 overexpression promoted the proliferation of cancer cells 15. Another study had pointed out that PRR14 was also high expressed in colorectal carcinoma, and its overexpression was related to tumor size and lymph node metastasis 16. Recent study had found that PRR14 overexpression was involved in breast cancer, and the cancer cell proliferation and tumor formation was promoted by its uprugulation 17. Moreover, as a proline rich protein, PRR14 may bind to the Src homology 3 (SH3) domain of Grb2 to activate PI3K signal in cancers 15, 17. However, the role and mechanism of PRR14 in cSCC was still no reported.

The study intends to reveal the expression of PRR14 in patients with cSCC, and the correlation of PRR14 expression with clinicopathological factors. Additionally, the effects of PRR14 expression on the proliferation, migration, invasion and apoptosis of cSCC cells were investigated through the activation of PI3K signal pathway. The results finally reveal the mechanism of PRR14 in the development and progress of cSCC, provide theoretical and experimental basis for cSCC gene therapy with PRR14 as a potential new target, and provide novel thinking and methods for clinical diagnosis and therapy of cSCC.

## Materials and methods

### Human specimens

The tumor tissues of patients with cutaneous squamous cell carcinoma were collected from the Affiliated Hospital of Nantong University (Nantong, China) between July 2013 and December 2016, meanwhile the corresponding para-carcinoma tissues were also collected as control. A total of 33 cases of fresh cSCC tumor and adjacent normal tissues were collected for mRNA and Western blot assay, 50 cases of paraffin-embedded cSCC tumor tissues were collected for immunohistochemistry. To be included in this study, patients who underwent surgical treatment without radiotherapy for the first time in Affiliated Hospital of Nantong University (Nantong, China) and were pathologically diagnosed with cSCC. Patients who with visceral cSCC skin metastasis and concomitant with other serious systemic diseases (such as chronic kidney disease, liver cirrhosis, heart failure or immune system defects) and other confirmed malignant tumors were excluded from the study.

### Cells and culture, siRNA and transfection

Human cSCC cell lines A431 and HSC-1, and normal skin cell line HaCaT obtained from the Institute of Cell Biology of Shanghai, Chinese Academy of Sciences (Shanghai, China), were cultured in Dulbecco's Modified Eagle's Medium (DMEM) (ThermoFisher Scientific, USA) containing 10% fetal bovine serum (FBS) (ThermoFisher Scientific, USA), and maintained in the 37 ˚C culture incubator with 5% CO_2_. The mRNA sequence of PRR14 gene was get from the NCBI database (NM_024031.5), and a PRR14 specific targeting small interfering RNA (siRNA) (siPRR14) was designed to silence endogenous PRR14 expression in cSCC cells, a scrambled siPRR14 sequence was designed and acted as siRNA negative control (siNC). The siRNAs were transfected to cells using Lipofectamine^®^3000 transfection reagent (ThermoFisher Scientific, USA) following the manufacturer's manual. The siRNAs and negative controls were synthesized by Biomics Biotechnologies Co., Ltd (Biomics Biotech, China) and the corresponding siRNA sequences were: siPRR14 sense, 5'-CAAUCAGGUUGAACAAGAAdTdT-3'; antisense, 5'-UUCUUGUUCAACCUGAUUGdTdT-3'; siNC sense, 5'-ACGAAUCAGAAUUACGAAGdTdT-3'; antisense, 5'-CUUCGTAAUUCUGAUUCGUdTdT-3.

### Immunohistochemical staining

The method of immunohistochemical staining was performed according to the previous study 18. Briefly, the paraffin-embedded tumor tissues were prepared into 4 μm tissue sections, and the sections were deparaffinized in xylene and rehydrated in concentration graded alcohol, then performed for the antigen retrieval using in 10 mmol/L boiled citrate buffer (pH 6.0) for 20 min, and endogenous peroxidase activities were blocked in 0.3% hydrogen peroxide solution for 10 min at 37 ˚C. Then, the sections were blocked using non-immune goat serum at room temperature for 20 min, and then incubated with the primary antibody anti-PRR14 (1:1,000 dilutions; #PA5-63828, ThermoFisher, USA) for 30 min at 37 ˚C followed 4 ˚C overnight. The sections were incubated with Goat Anti-Rabbit IgG H&L (horseradish peroxidase, HRP) (1:2,000 dilutions; #ab97051, Abcam, USA) for 30 min at room temperature after being washed in phosphate-buffered saline (PBS) (pH7.2), next to incubate in diaminobenzidine (DAB) solution for 3 min and then with hematoxylin staining. PRR14 immunostaining intensity was diagnosed by two pathologists independently. The staining intensity was graded as: 0 (negative staining), 1 (weakly positive staining), 2 (moderately positive staining) and 3 (strongly positive staining). The score of positive cell percentage was graded as: 0 (≤5%), 1 (6-25%), 2 (26-50%), 3 (51-75%) and 4 (>75%). The scoring results were calculated from staining intensity and score of positive cell percentage. PRR14 expression levels were defined to: “-” (negative staining, 0 score), “+” (weakly positive staining, 1-4 score), “++” (positive staining, 5-8 score), or “+++” (strongly positive staining, 9-12 score).

### Western blot analysis

Total proteins in cells or tissues were extracted with the pre-cold RIPA protein extraction buffer (Promega, USA) following the manufacturer's manuals, and quantified using bicinchonininc acid (BCA) method (Beyotime, China). Total of 40 μg proteins per lane were performed using sodium dodecyl sulfate (SDS)-polyacrylamide gel electrophoresis (PAGE), then transferred onto the polyvinylidene fluoride (PVDF) membranes (Merck-Millipore, USA). Subsequently, the membranes were blocked in 5% skim milk (BD, USA) for 2 h at room temperature, and then incubated with the primary each antibody separately, including PRR14 (1:500 dilutions; #SAB1102100, Merck, USA), phosphorylated (p)-PI3K (p-PI3K) (1:500 dilutions; #ab182651, Abcam, USA), PI3K (1:500 dilutions; #ab227204, Abcam, USA), p-AKT (1:500 dilutions; #ab38449, Abcam, USA), AKT (1:500 dilutions; #ab8805, Abcam, USA), p-mTOR (1:1,000 dilutions; #ab109268, Abcam, USA), mTOR (1:10,000 dilutions; #ab134903, Abcam, USA), andβ-actin (1:1000 dilutions; #ab8226, Abcam, USA) at 4 ˚C overnight. After being washed with tris-buffered saline (TBS) buffer containing 0.05% tween (TBST) for 5 min three times each, the membranes were incubated with goat anti-rabbit IgG H&L (HRP) (1:2,000 dilutions; #ab97051, Abcam, USA) for detection of PRR14, p-PI3K, PI3K, p-AKT, AKT, p-mTOR and mTOR, while rabbit anti-mouse IgG H&L (HRP) (1:2,000 dilutions; #ab97046, Abcam, USA) for β-actin at room temperature for 2 h. After being washed with TBST for 5 min three times each, the specific proteins were detected with ECL Substrate (ThermoFisher Scientific, USA), and then exposed to photographic films (Kodak, USA). The blots of films were quantified by ImageJ software (NIH, USA).

### Real-time quantitative PCR (RT-qPCR)

Total RNAs in cells or tissues were extracted using a TRIzol reagent (ThermoFisher Scientific, USA) following the manufacturer's manuals, and then submitted to a RT-qPCR reaction using One-Step SYBR-Green I qPCR kit (Biomics Biotech, China) following the manufacturer's protocols. β-actin was used as an internal normalized control. All amplified primers were designed and obtained from Biomics Biotech (China), and the sequences were: PRR14 forward, 5'-CAGGTTGAACAAGAAGGA-3' and reverse, 5'-CAAAGATGGTCTCAAAGGT-3'; β-actin forward, 5'-TGCACCACCAACTGCTTAGC-3' and reverse, 5'-GGCATGGACTGTGGTCATGAG-3'. The results were analyzed using previous reported 2^-ΔΔCt^ method 19.

### Cell proliferation assay

A Cell Counting Kit-8 (CCK-8) assay was used for the detection of cell proliferation. Briefly, 1.5×10^3^ cells were plated into 96-well plates per well and incubated cultured in the 37 ˚C culture incubator with 5% CO_2_, the cells were then transfected as above. Post-transfection for 24, 48 and 72 h, 100 μL PBS containing 10 μL CCK-8 working reagent was added per well, then incubated at 37 ˚C for 30 min. The optical density (OD) value of each well was measured at wavelength of 490 nm by a Microplate Reader machine (BioTek, USA). The data were analyzed and cell growth curve was drawn.

### Cell migration ability assay

A wound-healing assay was used for cell migration detection. In brief, 1×10^5^ cells per well were plated into 6-well plates and grown at 37 ˚C with 5% CO_2_ for 24 h. After being transfected with siRNAs for 4 h until the cell confluence reached about 70%, cell scratch wounds were made through confluent monolayer cells using a 1 mL pipette tip. Cell photos at different time 0 h, 24 h, 48 h, or 72 h were taken to observe cell migration ability. According to the cell growth at the scratch, the migration ability of the cells was calculated and analyzed.

### Cell invasive ability assay

A matrigel-based tanswell assay was used to determine cell invasion. Total of 1.5×10^5^ cells per well were plated onto 24-well plates and grown at 37 ˚C with 5% CO_2_ for 24 h. After being transfected with siRNAs for 48 h, the cells were resuspended in DMEM at a concentration of 5×10^5^ cells/mL. Transwell chambers with 8-μm pore size polycarbonate membrane (Corning, USA) were incubated in DMEM for 1 h before transfection, and then coated by 0.5 mg/mL Matrigel (BD, USA) at 37 ˚C for 4 h. Subsequently, 100 μL above cell suspension solution were added to upper chamber each, and 600 μL cell supernatant from un-transfection or transfection cells for 48 h was added to lower chamber each. After being incubating at 37 ˚C for 24 h, the cells on the top surface of the membrane were carefully removed using cotton swabs, and then the cells on the bottom surface of membrane were fixed using 10% formaldehyde for 30 sec. After being washed with PBS, the cells were stained using 0.5% crystal violet solution at room temperature for 30 min. After been washed in PBS, the cells on the bottom surface of the membrane were counted using a microscope in 3-5 fields randomly.

### Cell apoptosis assay

Annexin V-FITC and propidium iodide (PI) double-staining with flow cytometry was performed for cell apoptosis detection. In brief, 3×10^5^ cells per well were plated onto 6-well plates, then grown overnight at 37 ˚C with 5% CO_2_ for 24 h. Post 48 h transfection as above, the cells were collected and centrifuged for 5 min at 1,000 rpm, then resuspended in 195 μL 1×Binding Buffer (Sigma-Aldrich, USA), followed by 5 μL Annexin V-FITC solution (Sigma-Aldrich, USA) was added per sample and incubated at room temperature without lighting for 10 min. After being centrifuged at 1,000 rpm for 5 min, the cells were resuspended in 190 μL 1×Binding Buffer, followed by 10 μL PI (Sigma-Aldrich, USA) was added per sample. Subsequently, the stained cells were detected and analyze by a BD FACSCanto II flow cytometer (BD, USA).

### Statistical analysis

SPSS 20.0 and GraphPad Prism 7.0 softwares were used for data analysis. All data were showed as mean ± standard deviation (SD). A Student's t-test assay was used to analyze the difference between the two groups' data and one-way analysis of variance (ANOVA) followed by Tukey's post hoc test to analyze the difference between multiple groups. Pearson's correlation test was used for correlation analysis between PRR14 expression and clinicopathological factors. *P*<0.05 was considered as statistical significance.

## Results

### PRR14 is overexpressed in human cSCC patients and cells

**Figure 1:**
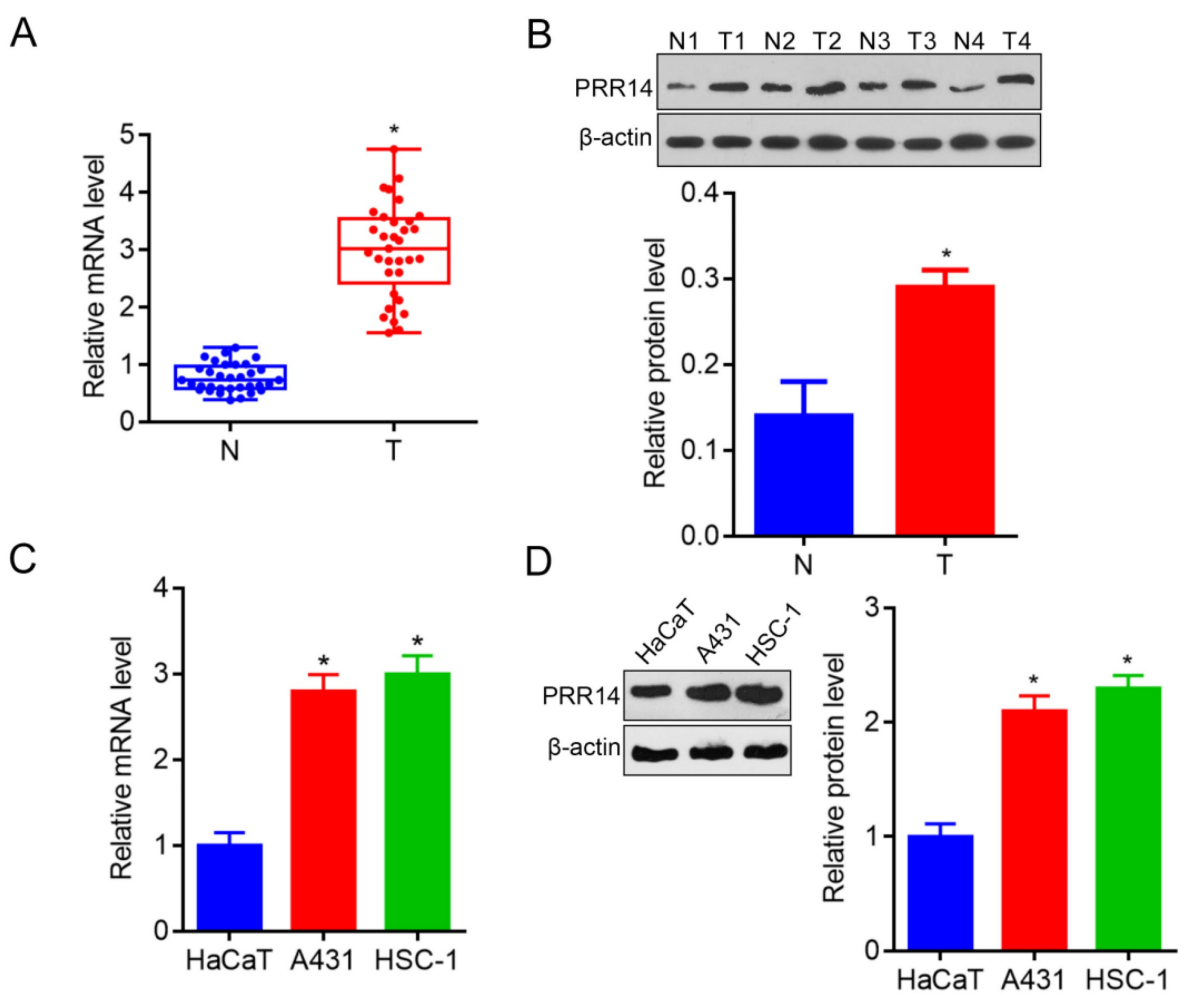
**PRR14 expressions in cSCC tissues and cells were detected by RT-qPCR.** (**A**) PRR14 mRNA levels in 33 cases of cSCC tissues and paired normal para-carcinoma tissues detected by RT-qPCR, *P<0.05, compared with that normal tissues. (**B**) PRR14 protein levels in cSCC tissues and paired normal para-carcinoma tissues detected by Western blot, *P<0.05, compared with that normal cutaneous tissues. (**C**) PRR14 mRNA levels in cSCC cell lines A431 and HSC-1 detected by RT-qPCR, *P<0.05, compared that in HaCaT cells. (**D**) PRR14 protein levels in cSCC cell lines A431 and HSC-1, and in HaCaT cells detected by Western blot, *P<0.05, compared that in HaCaT cells. N, normal cutaneous tissues; T, tumor tissues; N1, T1, the tissues from Patient 1; N2, T2, the tissues from Patient 3; N3, T3, the tissues from Patient 3; N2, T2, the tissues from Patient 4.

In order to explore PRR14 expression in human cSCC tissues, a total of 33 cases of fresh tumor tissues and para-carcinoma normal tissues were collected to investigate the PRR14 expression in cSCC patients using RT-qPCR and Western blot. To compare with that in normal tissues, PRR14 was overexpressed in cSCC tumor tissues both in the mRNA and protein levels (*P*<0.05) (Figure 1A and B). Additionally, A431 and HSC-1 cells were used to validate the PRR14 expression, the results showed that PRR14 mRNA and protein levels were upregulated in A431 and HSC-1 cSCC cells, compared with that in normal HaCaT cells (*P*<0.05) (Figure 1C and D).

### PRR14 expression correlated with clinicopathological factors in cSCC

**Figure 2:**
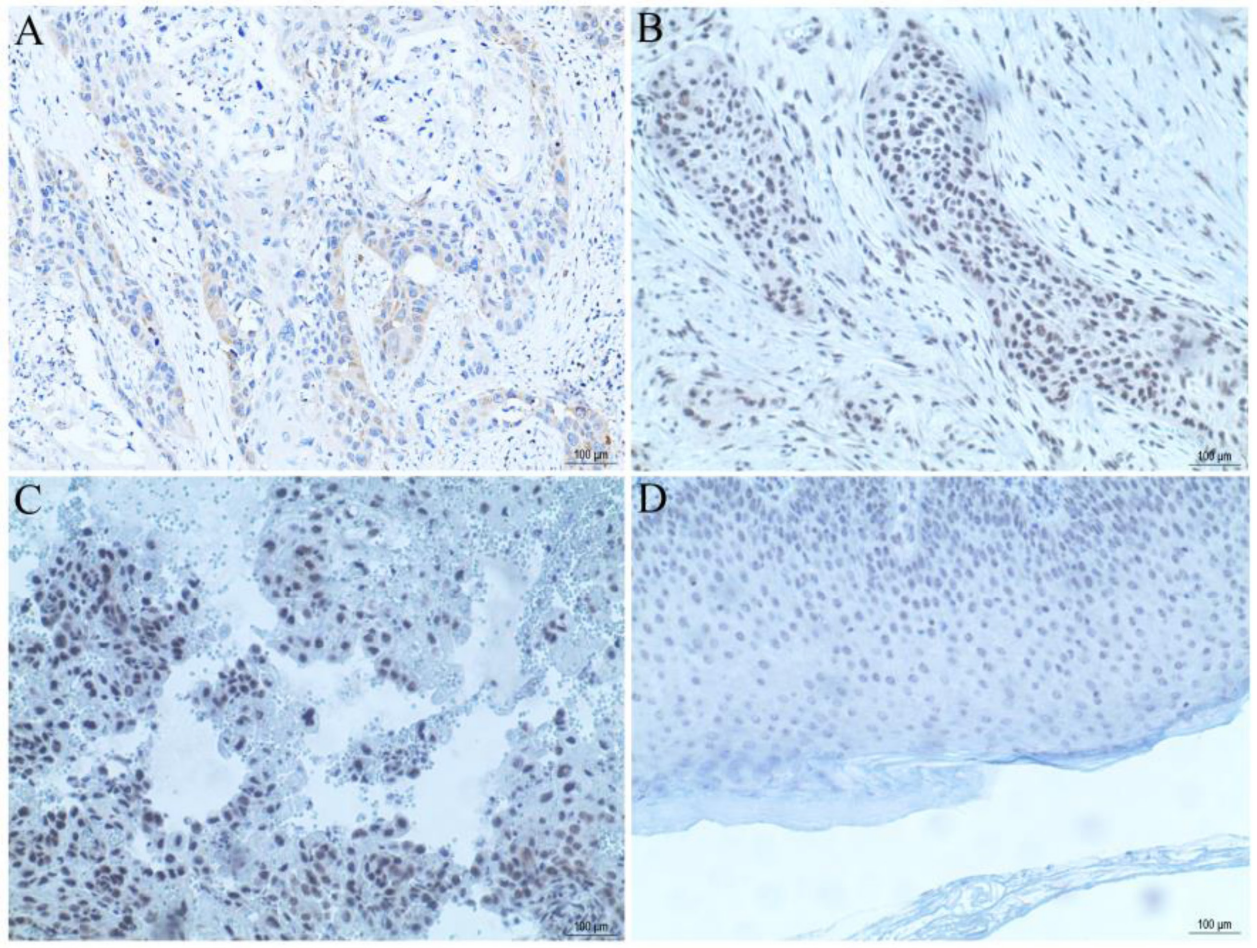
**PRR14 expression in cSCC tissues were analyzed by immunohistochemical staining.** (**A**) PRR14 expression in well-differentiated cSCC tissue. (**B**) PRR14 expression in moderately differentiated cSCC tissue. (**C**) PRR14 expression in poorly differentiated cSCC tissue. (**D**) PRR14 expression in normal cutaneous tissue. The blue staining indicates the nucleus, the yellow or brown staining indicates the PRR14 expression. (400×)

The tissue microarray section containing 50 cases of cSCC tissues was used for observation of PRR14 expression using immunohistochemical staining. The results showed that, there were 62% (31/50) moderate or strong PRR14 staining in cSCC tissues, 38% (19/50) weak or non-staining (Table 1, Figure 2), and negative staining in para-carcinoma normal tissues, indicated that PRR14 expression was higher in tumor tissues than in para-carcinoma tissues. Pearson's correlation analysis showed that, high expression of PRR14 was related to clinicopathological factors of cSCC patients, the significant differences were found between positive PRR14 expression with tumor thickness (*P*=0.036), TNM stage (*P*=0.040) or differentiation (*P*=0.024).

### PRR14 was effective silenced by RNA interfering in cSCC cells

**Figure 3:**
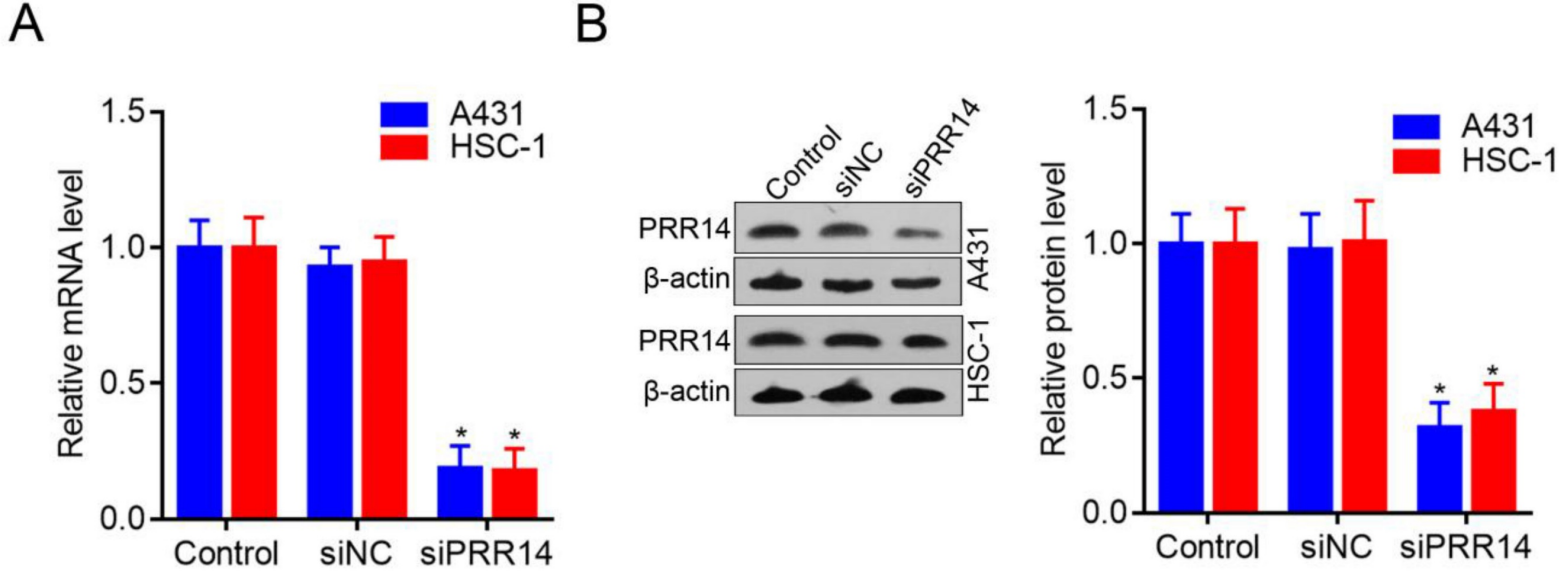
**PRR14 expression levels in cSCC cells and inhibited by siRNA.** (**A**) PRR14 mRNA levels inhibited by siPRR14 in A431 and HSC-1 cells. (**B**) PRR14 protein levels inhibited by siPRR14 both in A431 and HSC-1 cells. *P<0.05, compared with siNC treated cells.

To investigate the roles of PRR14 in cSCC cells, PRR14 specific-targeted siRNA (siPRR14) was designed to silence endogenous PRR14 expression levels. To compare with that in siNC treated cells, PRR14 mRNA levels were obvious downregulated by siPRR14 both in A431 and HSC-1 cells (*P*<0.05) (Figure 3A). Meanwhile, compared with that in siNC treated cells, PRR14 protein levels were also inhibited by siPRR14 significantly in A431 and HSC-1 cells (Figure 3B).

### Effects of PRR14 knockdown on cSCC cell growth

**Figure 4:**
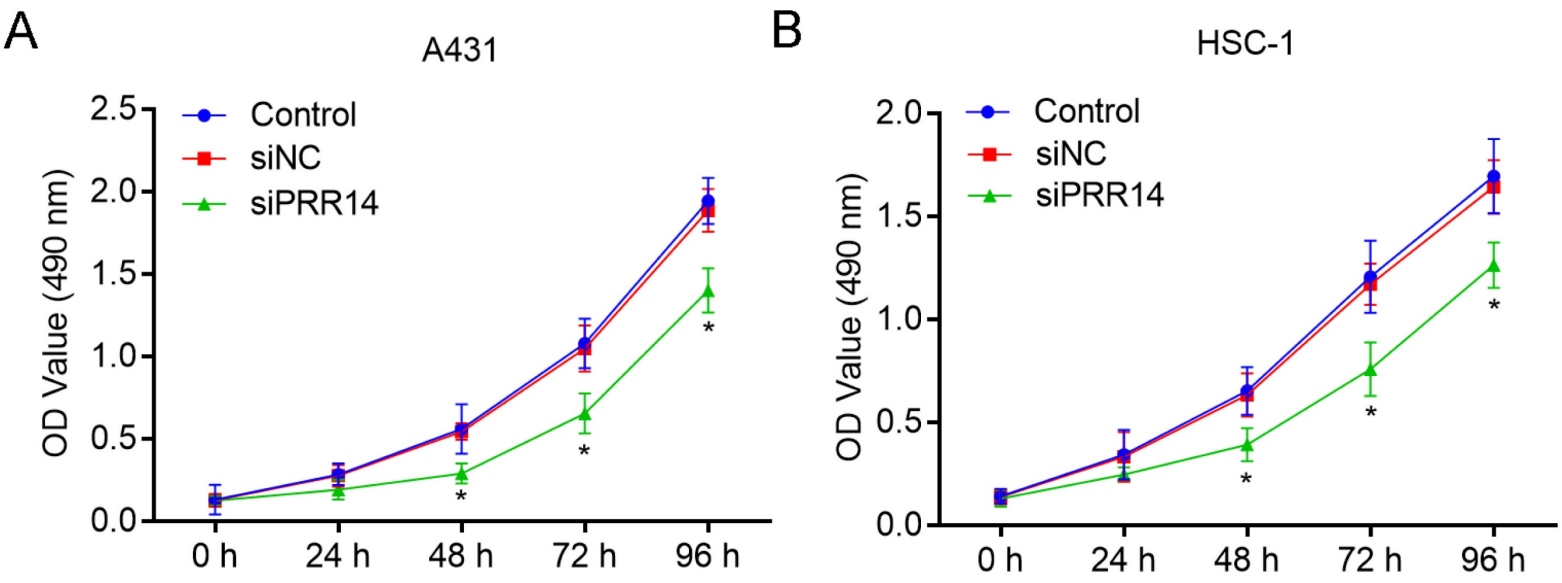
**Effects of PRR14 knockdown on the growth of cSCC cells.** (**A**) Effects of siPRR14 on A431 cells growth were detected by the CCK-8 assay. (**B**) Effects of siPRR14 on HSC-1 cells growth were detected by the CCK-8 assay. *P<0.05, compared with siNC treated cells.

The inhibitory effects of PRR14 knockdown on cSCC cell growth was detected using CCK-8 assay. To compare with siNC treated cells, siPRR14 inhibited the growth of A431 and HSC-1 cells at 48 h and 72 h significantly (*P*<0.05) (Figure 4).

### Effects of PRR14 knockdown on cSCC cell migration and invasion

**Figure 5:**
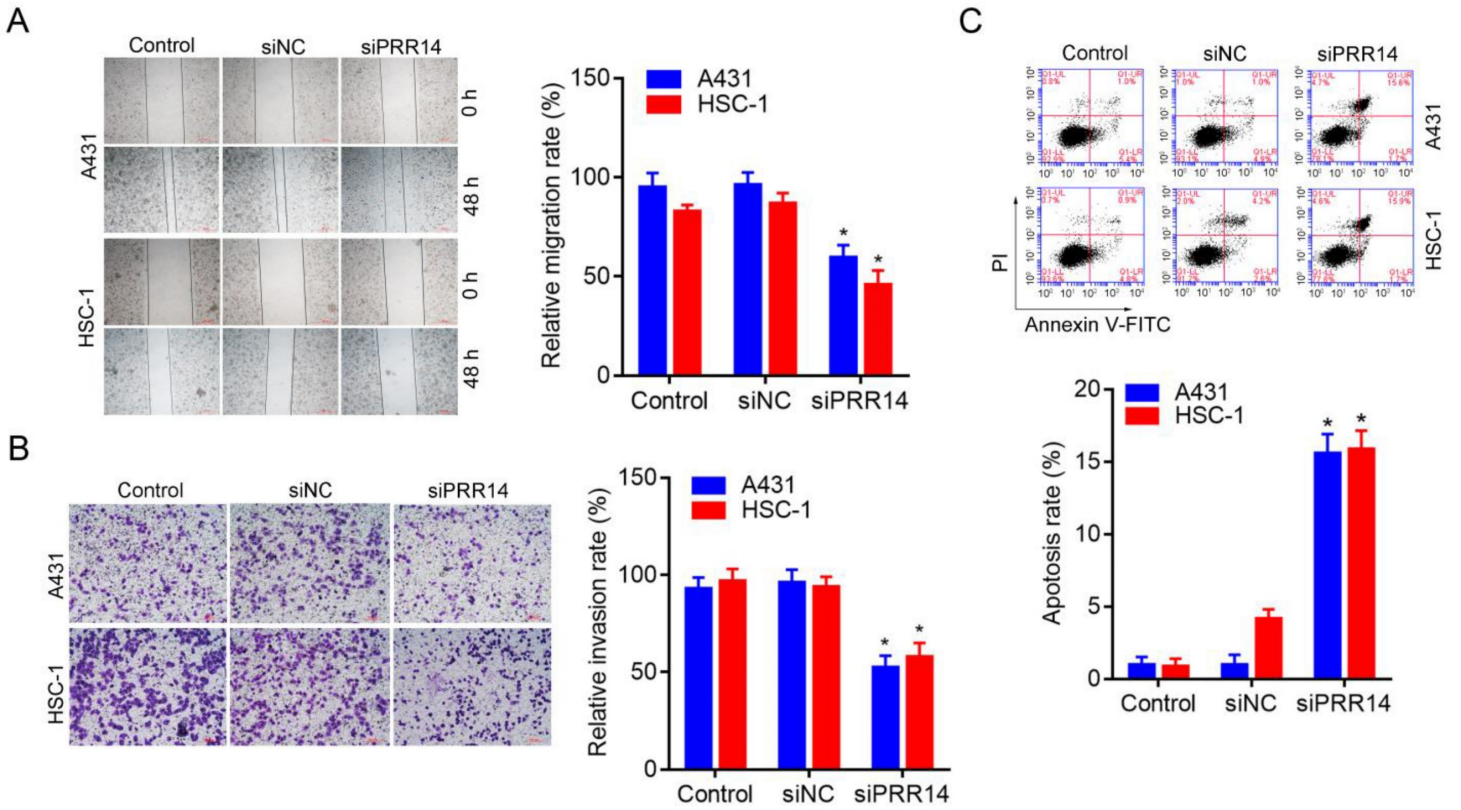
**Effects of PRR14 knockdown on the migration, invasion and apoptosis of cSCC cells.** (**A**) Effects of siPRR14 on A431 and HSC-1 cells migration were detected by the wound-healing assay. (**B**) Effects of siPRR14 on A431 and HSC-1 cells invasion were detected by matrigel based-transwell assay. (**B**) Effects of siPRR14 on A431 and HSC-1 cells apoptosis were determined by Annexin V-FITC/PI and flow cytometry assay. *P<0.05, compared with siNC treated cells.

A wound-healing assay was used to observe the inhibitory effect of PRR14 knockdown on cSCC cell migration, and a matrigel-based transwell assay was used to observe the inhibitory effect of PRR14 knockdown on cSCC cell invasion. The results showed that, siPRR14 suppressed the migration and invasion abilities of A431 and HSC-1 cell obviously, compared with that in siNC treated cells (*P*<0.05) (Figure 5A and B).

### Effects of PRR14 knockdown on cSCC cell apoptosis

The method of Annexin-V-FITC/PI double staining followed by flow cytometry was used to assess the inhibitory effect of siPRR14 on cSCC cell apoptosis. To compare with that in siNC treated cells, siPRR14 transfection for 48 h obvious promoted the apoptosis of A431 and HSC-1 cells (*P*<0.05) (Figure 5C).

### Effects of PRR14 knockdown on PI3K/Akt/mTOR signaling pathway

**Figure 6:**
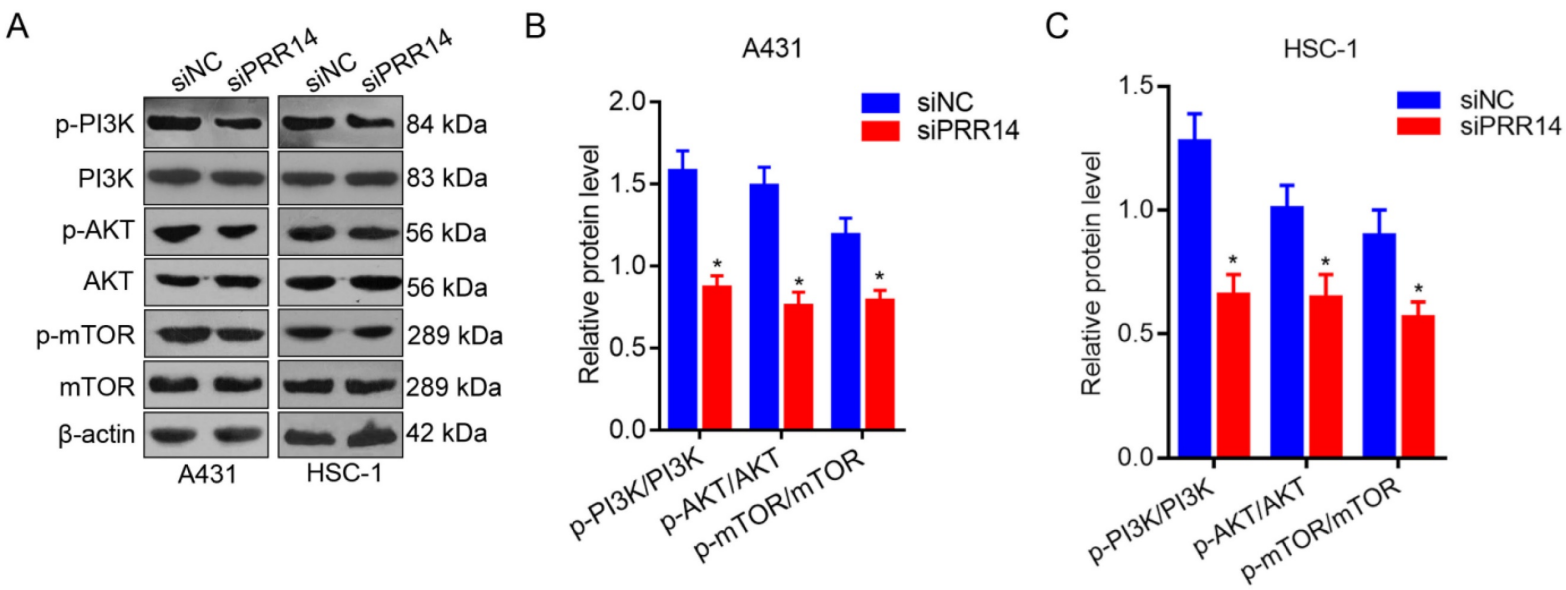
**Effects of PRR14 knockdown on PI3K/Akt/mTOR signaling pathway.** (**A**) The representative blots of p‑PI3K, PI3K, p‑AKT, AKT, p‑mTOR and mTOR proteins in siPRR14 treated A431 and HSC-1 cells. (**B**) The relative protein levels of p‑PI3K, PI3K, p‑AKT, AKT, p‑mTOR and mTOR in siPRR14 treated A431 cells. (**C**) The relative protein levels of p‑PI3K, PI3K, p‑AKT, AKT, p‑mTOR and mTOR in siPRR14 treated HSC-1 cells. *P<0.05, compared with siNC treated cells.

To further investigate the effects of PRR14 knockdown on PI3K/Akt/mTOR signaling pathway in cSCC cells. The phosphorylation levels of PI3K, AKT and mTOR were detected using Western blot method. To compare with that in siNC treated cells, the phosphorylation levels of PI3K, AKT and mTOR were upregulated in both siPRR14 treated A431 and HSC-1 cells significantly (*P*<0.05) (Figure 6).

## Discussion

Human cSCC is one of the malignant epithelial neoplasms with various inducing factors, but the pathogenesis and molecular mechanism are not clear yet 1, 20. With the rapid development of molecular biology, a variety of tumor-promoting genes has been discovered in cSCC, and their molecular mechanisms have been studied extensively.

Genomic analysis of cSCC has revealed many molecular mechanisms that promote the transformation of keratinocytes from benign to malignant, some mutated driver genes include TP53, which is the first tumor suppressor gene to be inactivated, and others such as the family members of neurogenic locus notch homolog protein 1 (NOTCH1), NOTCH2, cyclin-dependent kinase inhibitor 2A (CDKN2A), FAT atypical cadherin 1 (FAT1), phosphatidylinositol 3-kinase catalytic subunit alpha (PIK3CA), harvey rat sarcoma viral oncogene homolog (HRAS), epidermal growth factor receptor (EGFR), and phosphatase and tensin homolog deleted on chromosome ten (PTEN), which involved in cell-cycle regulation, cell differentiation, cell survival and proliferation 21.

In recent years, it has been found that a variety of novel molecules are involved in the occurrence and development of cSCC and as functions of promoting tumor formation through a variety of signaling pathways, such as apurinic/apyrimidinic endonuclease 1 (APE1) 22, DEAD-box helicase 46 (DDX46) 23, targeting protein for Xenopus kinesin-like protein 2 (TPX2) 24, kynureninase (KYNU) 25, signal transducer and activator of transcription 3 (STAT3) 26, histone deacetylase 3 (HDAC3) 27, SAM- and SH3-domain containing 1 gene (SASH1) 28, karyopherin subunit alpha 4 (KPNA4) 29, ephrin B receptor 2 (EphB2) 30, and cell division cycle 20 (CDC20) 31. APE1 has been reported to be significant high regulated in tumor tissues of human cSCC patients, and its high expression promoted the proliferation and migration of cSCC cells 22. DDX46 was obvious upregulated in human cSCC tissues and cells, it was found that DDX46 knockdown resulted in the suppression of cell proliferation, and the activation of cell apoptosis and autophagy, suggested DDX46 maybe a novel potential therapeutical target of cSCC 23. TPX2 was overexpressed in human cSCC patients, when silence of TPX2, the proliferation, migration, and invasion of cSCC cells were inhibited, while cell apoptosis was promoted through p53 signaling pathway 24. KYNU was overexpressed in cSCC patients and its knockdown suppressed the proliferation and metastasis ability of cSCC cells via PI3K/Akt signaling pathway 25. STAT3 maybe a novel marker in cSCC, and it downregulation inhibited cell proliferation but promoted cell apoptosis 26. It is reported that HDAC3 in the cSCC is up-regulated in cSCC, to compare with the normal dermal tissue, and its inhibitor Rg3 treatment inhibited the epithelial mesenchymal transformation (EMT) in cSCC cell, indicated that HDAC3 maybe serve as a target for cSCC therapy 27. Instead, SASH1 expression was significant decreased in cSCC cells, and its high expression suppressed the viability and migration of cSCC cells, and the inhibition effects maybe through inhibition of Akt signal 28. KPNA4 has been reported to be overexpressed in cisplatin-resistant cSCC cells, and it inhibited by miR-3619-5p resulted in the suppression of cell proliferation and cisplatin resistance of cSCC 29. EphB2 may be a therapeutical target to promote of the migration and invasion of cSCC cells and tumor angiogenesis, and *in vitro* and* in vivo* experiment both showed that EphB2 inhibition obvious suppressed the cell tumorigenesis, while inhibited cell apoptosis and altered the cell cycle 30. CDC20 maybe an oncogenic role in patients with primary cSCC, and its expression was significant upregulated in cSCC which correlated with tumor differentiation. CDC20 decrease inhibited cell proliferation and migration, induced cell cycle arrest but promoted cell apoptosis through Wnt/β-catenin signaling pathway 31. In the study, we confirmed a novel oncogene PRR14 in cSCC. PRR14 has found to be a novel biomarker for some human cancers 15-17, but there is no report in human cSCC. We first found that PRR14 expression was overexpressed in tumor tissues from cSCC patients in this study, and found that its overexpression was related to clinicopathological factors including tumor thickness, TNM stage and differentiation.

In addition, PRR14 has been reported to be overexpressed in patients with NSCLC, and further results showed that PRR14 may be a new activator of PI3K/Akt signaling pathway in NSCLC development. As a proline rich protein, PRR14 may bind to the Src homology 3 (SH3) domain of Grb2 to activate PI3K signal, suggests that PRR14 may be used as a prognostic factor independently and a potential target for NSCLC treatment 15. Also, PRR14 was overexpressed in colorectal cancer, and functional studies shown that PRR14 suppression can inhibit the proliferation, migration and invasion of colon cancer cells though Akt signaling pathway 16. Moreover, PRR14 has found to be high expressed in breast cancer, and promoted cell proliferation by activating PI3K/Akt/mTOR pathway 17. However, there is no evidence for the molecular mechanism of PRR14 in cSCC.

In the study, we explored that PRR14 was also high expressed in cSCC cell lines A431 and HSC-1, and the proliferation, migration and invasion of cSCC cells were inhibited by PRR14 knockdown, while cell apoptosis was promoted. On the contrary, when PRR14 was inhibited by siRNA, the proliferation, migration and invasion of cSCC cells were suppressed, while cell apoptosis was promoted. Further study also confirmed that the phosphorylation levels of PI3K, Akt and mTOR were upregulated after PRR14 knockdown in A431 and HSC-1 cells.

In summary, we first explored that PRR14 was high expressed in human cSCC, and its overexpression was associated with tumor thickness, TNM stage and differentiation, suggesting that PRR14 might be a potential poor prognosis factor for cSCC; and the activator role of PRR14 in cSCC carcinogenesis maybe partial through PI3K/Akt/mTOR signal pathway, suggesting that PRR14 might be a new therapeutic target for cSCC treatment.

## Figures and Tables

**Table 1:** Correlation between PRR14 expression and clinicopathological factors in cSCC.

Clinicopathological factors	Cases no.	PRR14 expression, n			*Pearson x^2^*	*P*
		-/+	++	+++		
Gender					0.523	0.770
Male	21	7	8	6		
Female	29	12	11	6		
Age (years)					2.047	0.359
≤60	5	3	2	0		
>60	45	16	17	12		
Thickness					6.632	0.036*
≤5 mm	24	10	12	2		
>5 mm	26	9	7	10		
TNM stage					13.181	0.040*
I	3	3	0	0		
II	26	11	12	3		
III	18	5	5	8		
IV	3	0	2	1		
Differentiation					11.284	0.024*
Well	20	7	10	3		
Moderately	23	12	5	6		
Poorly	7	0	4	3		
Site					0.658	0.720
Sunlight exposure	2	1	1	0		
Non exposure	48	18	18	12		

*Represents statistical significance, *P*<0.05

## Abbreviations

PRR14: proline rich protein 14
cSCC: cutaneous squamous cell carcinoma
IHC: immunohistochemistry
RT-qPCR: real-time quantitative PCR
CCK-8: cell counting kit-8
TNM: tumor node metastasis
RNAi: RNA interfering
mTOR: mammalian target of rapamycin
PI3K: phosphoinositide 3-kinase
NLS: N-terminal nuclear localization signals
NSCLC: non-small cell lung cancer
DMEM: Dulbecco's Modified Eagle's Medium
FBS: fetal bovine serum
NC: negative control
siRNA: small interfering RNA
HRP: horseradish peroxidase
PBS: phosphate-buffered saline
DAB: diaminobenzidine
BCA: bicinchonininc acid
SDS: sodium dodecyl sulfate
PAGE: polyacrylamide gel electrophoresis
PVDF: polyvinylidene fluoride
TBS: tris-buffered saline
PI: propidium iodide
SD: standard deviation
ANOVA: analysis of variance
NOTCH: neurogenic locus notch homolog protein 1, homolog protein
CDKN2A: cyclin-dependent kinase inhibitor 2A
FAT1: FAT atypical cadherin 1
PIK3CA: phosphatidylinositol 3-kinase catalytic subunit alpha
HRAS: harvey rat sarcoma viral oncogene homolog
EGFR: epidermal growth factor receptor
PTEN: phosphatase and tensin homolog deleted on chromosome ten
APE1: apurinic/apyrimidinic endonuclease 1
DDX46: DEAD-box helicase 46
TPX2: targeting protein for Xenopus kinesin-like protein2
KYNU: kynureninase
STAT3: signal transducer and activator of transcription 3
HDAC3: histone deacetylase 3
SASH1: SAM- and SH3-domain containing 1 gene
KPNA4: karyopherin subunit alpha 4
EphB2: ephrin B receptor 2
CDC20: cell division cycle 20
EMT: epithelial mesenchymal transformation
SH3: Src homology 3
